# Evaluation of procalcitonin versus conventional inflammatory biomarkers for clinical severity grading in patients with intra-abdominal infection

**DOI:** 10.1007/s00423-025-03636-5

**Published:** 2025-03-11

**Authors:** Cihan Ozen, Deniz Karasoy, Ali Yalcinkaya, Sine Huus Pedersen, Steen Kaare Fagerberg, Peter Hindersson, Peter Derek Christian Leutscher, Kathrine Holte

**Affiliations:** 1 Department of Gastrointestinal Surgery, Aalborg University Hospital, Aalborg, Denmark; 2 Department of Medicine, Holbaek Hospital, Medicine 1, Holbaek, Denmark; 3 Department of Clinical Medicine, Faculty of Medicine, Aalborg University, Aalborg, Denmark; 4 Department of Pathology, North Denmark Regional Hospital, Hjoerring, Denmark; 5 Department of Clinical Diagnostic, North Denmark Regional Hospital, Hjoerring, Denmark; 6 Centre for Clinical Research, North Denmark Regional Hospital, Hjoerring, Denmark; 7 Department of Gastrointestinal Surgery, North Denmark Regional Hospital, Hjoerring, Denmark

**Keywords:** Procalcitonin, Intra-abdominal infection, Inflammatory biomarkers

## Abstract

**Aim:**

We aimed to evaluate the utility of procalcitonin (PCT) as a biomarker for clinical severity grading of intra-abdominal infections (IAI) in hospital-admitted patients presenting with acute abdomen.

**Methods:**

In this retrospective study, median PCT values were compared with conventional inflammatory biomarkers, including leukocyte count (LC), neutrophil count (NC), and C-reactive protein (CRP), within the patient population.

**Results:**

Among the 245 patients included in the study, 58 (23.7%) were diagnosed with appendicitis, 54 (22.0%) with diverticulitis, 34 (13.9%) with calculous cholecystitis, and 21 (8.6%) with pancreatitis. Additionally, 60 (24.5%) were diagnosed with non-specific abdominal pain (NSAP), and 18 (7.3%) with gallstones without cholecystitis. Median PCT levels were significantly higher in patients with calculous cholecystitis (*p* < 0.0001) and pancreatitis (*p* < 0.0001) compared to those with NSAP. The proportion of patients with a PCT cut-off ≥ 0.04 µg/L was significantly higher across all IAI subgroups compared to the NSAP group. However, 18 (10.8%) of IAI patients exhibited PCT levels ≥ 0.5 µg/L, indicating systemic infection. Spearman’s rho analysis revealed a significant correlation between PCT and LC, NC, and CRP in patients with IAI (*p* < 0.0001). Moreover, median PCT levels were significantly higher in perforation/abscess vs. gangrenous appendicitis (*p* < 0.01), complicated vs. uncomplicated diverticulitis (*p* = 0.048), and severe vs. mild cholecystitis (*p* < 0.001).

**Conclusion:**

PCT correlates strongly with conventional inflammatory biomarkers in patients with IAI. However, PCT appears to offer limited additional clinical value for guiding therapeutic decisions concerning the initial diagnosis and/or severity grading of IAI in patients admitted with acute abdomen. Further research is warranted to validate these findings.

## Introduction

Intra-abdominal infection (IAI) is a common cause of acute abdomen, often necessitating prompt clinical evaluation. In severe cases, antibiotic therapy may be required to prevent complications such as peritonitis and septicemia [1, 2]. IAI encompasses several common surgical emergency diagnoses, including appendicitis, cholecystitis, and diverticulitis. Specific antibiotic guidelines exist for various IAI diagnoses, with clinical severity grading being crucial for determining the appropriate antibiotic treatment. However, not all IAI cases, such as uncomplicated diverticulitis [3] and mild pancreatitis [4], require antibiotics.

Conventional inflammatory biomarkers, including leukocyte count (LC), neutrophil count (NC), and C-reactive protein (CRP) [5], are routinely utilized to assist in diagnosing patients with suspected IAI and acute abdomen [6]. Elevated levels of these biomarkers often indicate bacterial infections that may necessitate antibiotic therapy [7]. However, the effectiveness of these conventional inflammatory biomarkers in guiding antibiotic treatment decisions warrants further investigation [8]. While these biomarkers remain valuable in acute settings, they lack diagnostic accuracy for complicated appendicitis [9, 10].

In recent years, plasma procalcitonin (PCT) has gained increasing attention as a laboratory test for detecting and monitoring bacterial infections of various origins [11, 12]. Although PCT levels are typically very low (< 0.1 ng/mL) in healthy individuals, they rise in response to bacterial infections, making PCT a more specific inflammatory marker of bacterial disease compared to CRP [13]. The standard PCT cut-off value for bacterial infection is 0.5 ng/mL (µg/L), with a reported sensitivity of 76% and specificity of 69% [14]. Although PCT has been reported to correlate with the clinical severity of appendicitis [15–18], cholecystitis [19, 20], diverticulitis [21], and pancreatitis [22, 23], its effectiveness as a biomarker for diagnosing IAI in patients with acute abdomen, compared to conventional inflammatory biomarkers, remains unclear [24–26].

This study aimed to evaluate procalcitonin (PCT) in comparison with conventional inflammatory biomarkers (LC, NC, and CRP) for severity grading of intra-abdominal infection (IAI) diagnoses among patients admitted to the surgical emergency department with acute abdomen.

## Materials and methods

### Data sources

Demographic and clinical data were retrieved from the patient’s digital medical records, including age, gender, biochemistry with a focus on conventional inflammatory biomarkers and PCT, radiological/histopathological findings, and diagnoses based on the International Classification of Diseases, 10th Revision (ICD-10) [27]. The REDCap data management system was utilized for the collection and management of the study data [28].

### Study population

This single-center retrospective study was conducted at the North Denmark Regional Hospital. The study population consisted of adult patients (age ≥ 18 years) with acute abdomen admitted to the surgical emergency department during a four-month period between 01/05/2018 and 31/08/2018. Patients who received antibiotics within the previous 4 weeks prior to admission were excluded. The population was divided according to the final diagnosis, either non-specific abdominal pain (NSAP) or one of the following four IAI diagnoses (i.e., acute appendicitis, diverticulitis, calculous cholecystitis, or pancreatitis).

### Clinical severity grading of IAI

Each of the four specific IAI diagnoses was subcategorized by clinical severity according to radiological and histopathological findings. The diagnosis of acute appendicitis (phlegmonous, gangrenous, or perforation/abscess) [16] was based on the extent of inflammation in the appendix and determined through a combination of intraoperative observations and both macroscopic and microscopic evaluations of the removed appendix. Phlegmonous appendicitis featured inflammatory cell infiltration while maintaining the structural integrity of the appendix; gangrenous appendicitis involved inflammatory cell infiltration accompanied by damage to the structural architecture but lacked abscess formation or perforation; severe appendicitis was characterized by significant inflammatory cell infiltration, architectural destruction, and the presence of either a visible peri-appendiceal abscess or perforation. The diagnosis of calculous cholecystitis (mild or severe) [29] was evaluated by combining ultrasound and computed tomography with Paraclinical values, including fever, and histopathology results. Patients with diverticulitis (uncomplicated or complicated—classified according to Hinchey 1–2 and 3–4 classifications) were evaluated using abdominal tomography based on the Hinchey classification [30]. Uncomplicated cases of diverticulitis (Hinchey 0) were not treated with antibiotics, whereas complicated cases (Hinchey 1–2) received either antibiotics, drainage treatment, or both. For those with complicated (Hinchey 3–4) diverticulitis, surgery was performed along with the previously mentioned treatments. During the surgery, either laparoscopic intraoperative lavage or the Hartmann procedure was utilized. Pancreatitis patients were divided into mild and severe according to the Atlanta classification [31] based on abdominal tomography results. The patients were also evaluated according to the Glascow-Imri score [32] to determine organ failure and antibiotic treatment was initiated for patients scoring 2 or more. It is important to note that patients with a final diagnosis of gallstones without cholecystitis were not considered as having an IAI diagnosis.

### Inflammatory biomarkers

Blood samples for routine biochemical and hematological analyses, including conventional inflammatory biomarkers (LC, NC, and CRP), were obtained from patients within 2 h of admission to the surgical emergency department. The results of the laboratory analyses were available for clinical evaluation within one hour. In contrast, PCT was measured in a batch of pooled plasma samples after the study inclusion period ended, ensuring that the attending clinicians were blinded to the PCT results upon admission. The laboratory reference values for the three conventional inflammatory biomarkers were as follows: LC (≥ 10.0 × 10⁹/L), NC (≥ 7.00 × 10⁹/L), and CRP (≥ 8.0 mg/L). A laboratory cut-off value of ≥ 0.04 µg/L was used in this study, in accordance with local standard operating procedures at the Department of Medical Biochemistry, North Denmark Regional Hospital. A PCT cut-off value of ≥ 0.5 µg/L [33, 34] is typically considered indicative of systemic infection.

### Statistical methods

Nominal and ordinal parameters were described as percentages using frequency analysis. Fisher’s exact test was employed to assess differences between categorical variables. Continuous variables were tested for normality, and non-parametric analyses were used based on the distribution; therefore, they are described using median values with interquartile ranges (IQR). The conventional inflammatory biomarkers and PCT were analyzed within the IAI diagnoses and compared to the values obtained from patients diagnosed with NSAP. The two-sample Wilcoxon rank-sum test (Mann-Whitney U test) was used to assess the equality of medians, while the Kruskal-Wallis test was utilized to evaluate the equality of medians across three or more subgroups. Spearman’s rho correlation analysis was conducted to analyze the correlation between conventional inflammatory biomarkers and PCT among patients with IAI diagnoses, with log-transformed values of CRP and PCT presented for better illustration of distribution patterns.

Sensitivity, specificity, positive predictive value (PPV), and negative predictive value (NPV) were calculated for each biomarker, including PCT, LC, CRP, and NC, to assess their diagnostic accuracy in intra-abdominal infections. All statistical tests were two-sided, and a p-value of < 0.05 was considered the threshold for statistical significance. Statistical analyses were performed using Stata version 17.

### Ethical considerations and Project Registration

Approval from the North Denmark Regional Committee for Health Research Ethics was not deemed necessary, as this was a clinical quality assurance study. The study was registered in the Clinical Research Registry of the North Denmark Region.

## Results

A total of 245 patients admitted with a surgical emergency were included in the study (Table 1). The median age was 53 years (IQR 38–67), and 146 patients (59.6%) were female. Among these, 167 patients (68.2%) were diagnosed with one of four specified intra-abdominal infections (IAIs): acute appendicitis (*n* = 58, 23.7%), calculous cholecystitis (*n* = 34, 13.9%), diverticulitis (*n* = 54, 22.0%), and pancreatitis (*n* = 21, 8.6%). Additionally, 60 patients (24.5%) presented with non-specific abdominal pain (NSAP), while 18 patients (7.3%) had gallstones without associated cholecystitis.

**Table 1:** Demographic and clinical characteristics of the study population

	*N* = 245
Age, years, median (IQR)	53 (38–67)
Gender, *n* (%)	
Females	146 (59.6)
Males	99 (40.4)
Diagnoses, *n* (%)	
Acute appendicitis	58 (23.7)
Phlegmonous	19 (7.8)
Gangrenous	23 (9.4)
Perforation/abscess	16 (6.5)
Diverticulitis	54 (22.0)
Uncomplicated	37 (15.1)
Complicated	17 (6.9)
Gallstone ± cholecystitis	52 (21.2)
Gallstones without cholecystitis	18 (7.3)
Mild calculous cholecystitis	20 (8.2)
Severe calculous cholecystitis	14 (5.7)
Pancreatitis	21 (8.6)
Mild	18 (7.4)
Severe	3 (1.2)
Non-specific abdominal pain	60 (24.5)

IQR:25th– 75th Interquartile range

For those diagnosed with diverticulitis (*n* = 54), CT scans were employed as the primary diagnostic tool. Within this cohort, 37 patients exhibited uncomplicated diverticulitis, whereas 17 presented with complicated diverticulitis as per the Hinchey classification. Specifically, 12 patients were classified within the Hinchey 1–2 category and 5 patients within the more severe Hinchey 3–4 category.

All patients in the Hinchey 3–4 group required surgical intervention. One case involved severe diverticulitis complicated by stenosis, for which a transversostomy was performed. In two cases, intra-abdominal abscesses were detected and managed with peritoneal lavage and drainage. The remaining two patients had perforated diverticulitis with peritonitis, necessitating sigmoid resection and a Hartmann colostomy.

All 21 patients diagnosed with pancreatitis underwent CT scans and either ultrasound or MRI scans. Among the 18 patients with mild pancreatitis, the identified causes were: gallstones in 4 cases, excessive alcohol use in 5, suspected pancreatic cancer in 1, and 8 cases with no identified cause upon emergency room assessment. Of the 3 patients with necrotic pancreatitis, 2 had a history of heavy alcohol use, while the cause remained unclear for the third patient.

### Comparison of PCT and conventional inflammatory biomarkers

Table 2 presents the median values (IQR) of the conventional inflammatory biomarkers (LC, NC, CRP) and PCT, comparing the NSAP group with the four IAI subgroups. Additionally, it compares the number (%) of patients with PCT cut-off values ≥ 0.04 µg/L and ≥ 0.5 µg/L, respectively. We found that all IAI diagnoses exhibited significantly increased values of the conventional inflammatory biomarkers compared to the NSAP group. The median PCT values were significantly higher in patients with cholecystitis and pancreatitis compared to those with NSAP. While the median PCT was comparable to that of NSAP for acute appendicitis and diverticulitis, the proportions of patients with PCT cut-off values ≥ 0.04 µg/L were significantly higher in all four IAI subgroups compared to NSAP. A similar trend was observed for PCT cut-off values ≥ 0.5 µg/L, except in patients with diverticulitis, where too few patients in all IAI diagnoses exhibited PCT cut-off values ≥ 0.5 µg/L.

**Table 2:** Inflammatory biomarkers findings in patients with non-specific abdominal pain versus patients with appendix, diverticulitis, calculous cholecystitis, and pancreatitis, respectively

		Median values (IQR)				*n* (%)	
		Leukocyte count	Neutrophile count	C-reactive protein	Procalcitonin	Procalcitonin*	
	*n*	10^9^/L	10^9^/L	mg/L	µg/L	≥ 0.04 µg/L	≥ 0.5 µg/L
Non-specific abdominal pain	60	9.1 (7.3–11.1)	6.2 (4.7–7.7)	5.5 (2.9–15.5)	0.05 (0.05–0.07)	17 (28.3)	0 (0)
versus							
Acute appendicitis	58	13.2 (10.5–17.5)	11.3 (7.8–14.5)	37.5 (9.9–77.0)	0.06 (0.04–0.12)	27 (46,6)	5 (8.6)
*p-value*		< 0.0001	< 0.0001	< 0.0001	0.06	< 0.01	< 0.01
Diverticulitis	54	11.4 (9.3–13.3)	8.6 (6.5–10.3)	82.2 (48.0–121.0)	0.05 (0.04–0.1)	22 (40.7)	1 (1.9)
*p-value*		0.0001	< 0.0001	< 0.0001	0.12	0.02	0.23
Calculous cholecystitis †	34	13.4 (10.6–17.2)	10.4 (8.1– 14.5)	81.5 (13.0–177.0)	0.27 (0.05–0.5)	22 (64.7)	6 (17.6)
*p-value*		< 0.0001	< 0.0001	< 0.0001	< 0.0001	< 0.0001	< 0.0001
Pancreatitis	21	12.3 (10.1–14.7)	9.3 (7.3–12.7)	74.6 (7.8–94)	0.24 (0.07–0.71)	13 (61.9)	6 (28.6)
*p-value*		< 0.0001	< 0.0001	< 0.001	< 0.0001	< 0.001	< 0.0001

IQR: 25th– 75th Interquartile range

Two-sample Wilcoxon rank-sum (Mann-Whitney) test was applied to assess the equality of medians comparing NSAP with IAI diagnoses

* Fisher´s exact test was used to assess the differences for the categorical variables

† Calculous cholecystitis excluding gallstones without cholecystitis

**Fig. 1:**
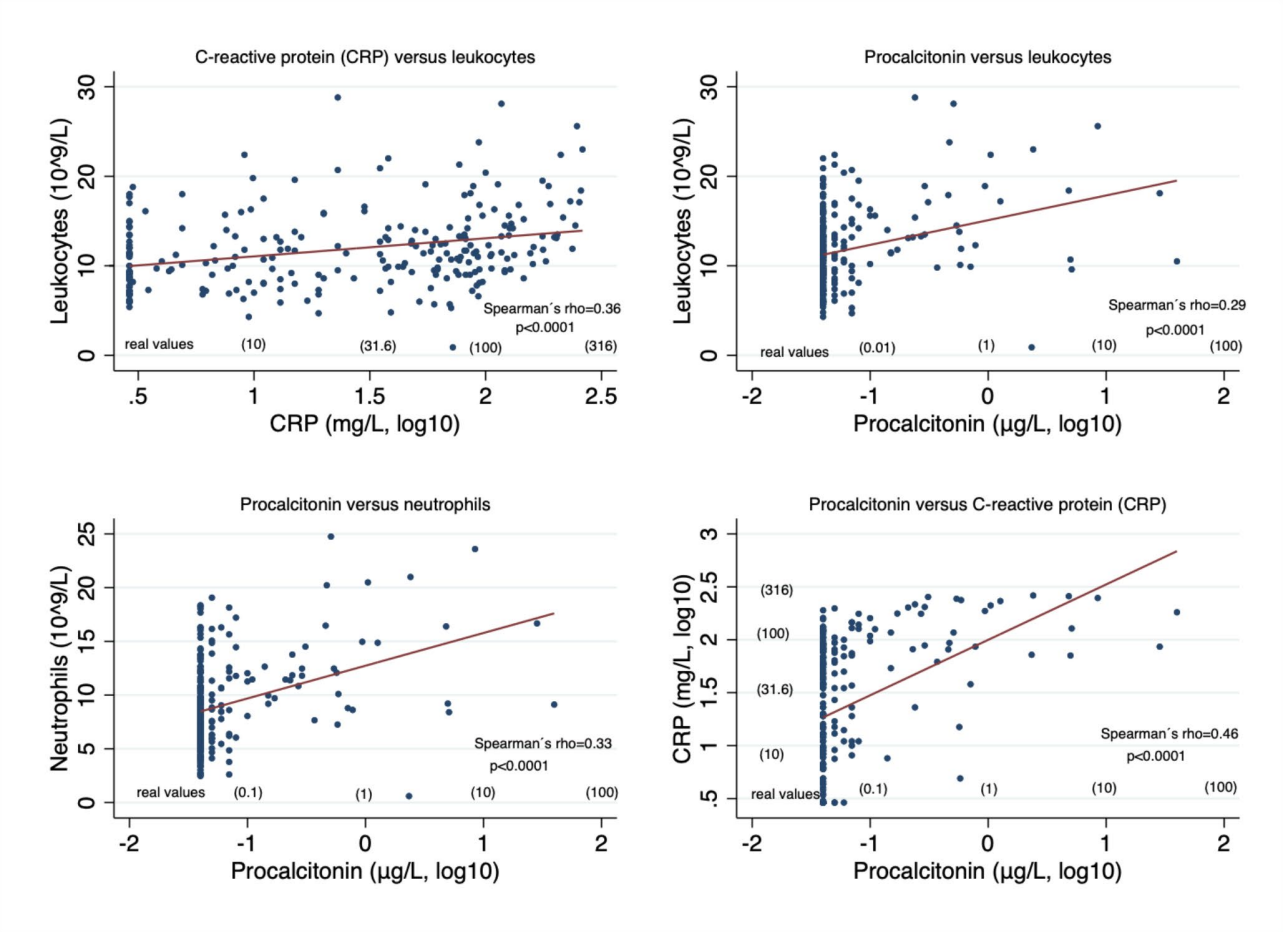
Spearman’s rho correlation analyses between the inflammatory biomarkers in patients with intra-abdominal infections

Figure 1 depicts Spearman’s rho correlation analyses among conventional inflammatory biomarkers and PCT in patients with IAI diagnoses. All conventional inflammatory biomarkers and PCT exhibited significant correlations in IAI patients; LC vs. CRP (*p* < 0.0001), LC vs. PCT (*p* < 0.0001), NC vs. PCT (*p* < 0.0001), and CRP vs. PCT (*p* < 0.0001), respectively.

### Biomarkers in a clinical severity grading context

**Fig. 2:**
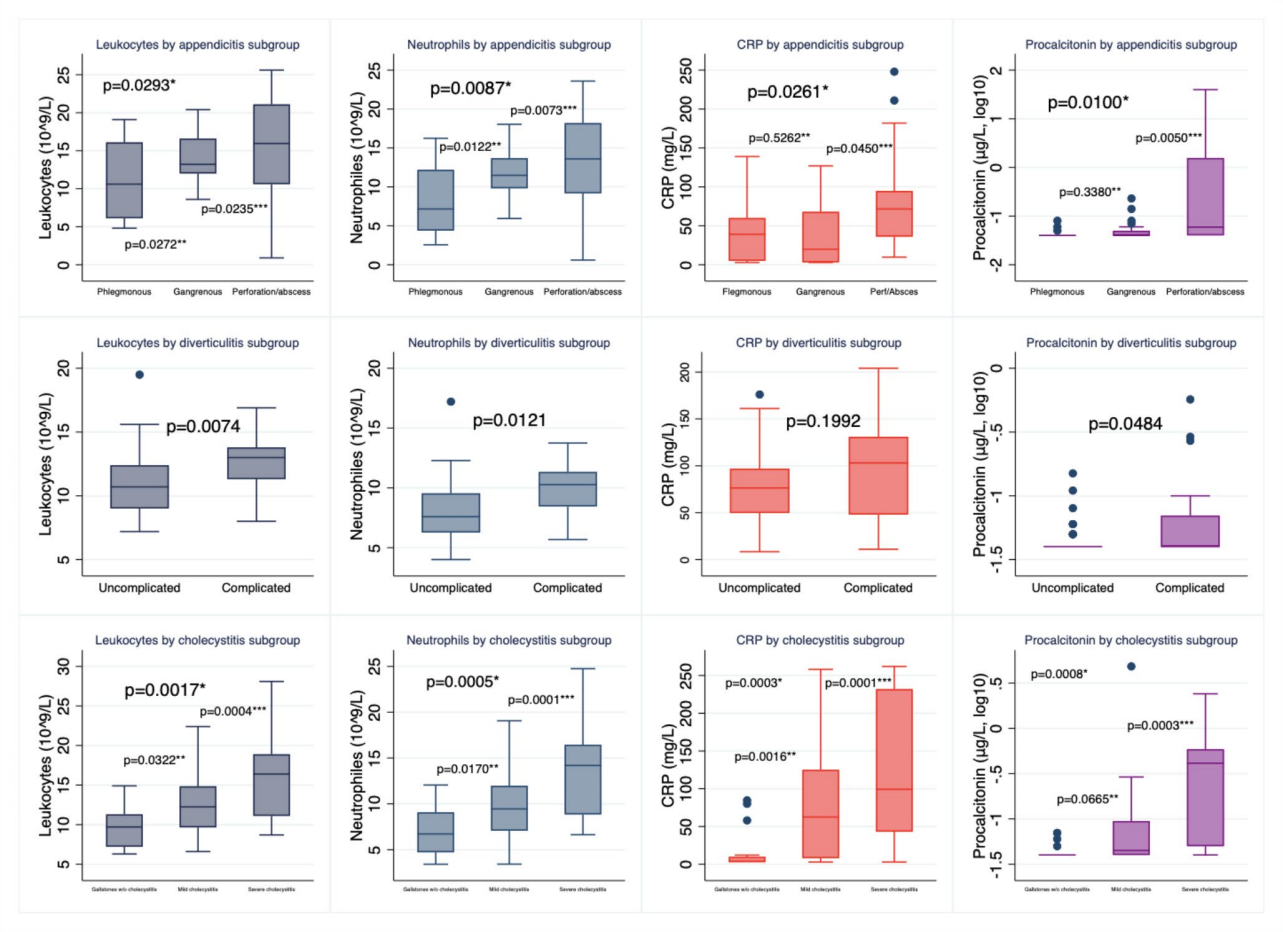
Comparison of inflammatory biomarkers’ median (IQR) by clinical severity of the intra-abdominal infections. *=Kruskall-Wallis test. **=Mann-Whitney U test between gangrenous appendicitis/mild cholecystitis and phlegmonous appendicitis/gallstone without cholecystitis, respectively. ***=Mann-Whitney U test between perforated appendicitis/severe cholecystitis and phlegmonous appendicitis/gallstones without cholecystitis, respectively

Figure 2 illustrates the comparative representation of the conventional inflammatory biomarkers and PCT according to the severity of appendicitis, diverticulitis, and cholecystitis. More severe cases of appendicitis exhibited significantly and consistently higher LC and NC median values. Although we observed significantly different medians of CRP (*p* = 0.026) and PCT (*p* = 0.01) among appendicitis subgroups, CRP and PCT medians showed no significant difference between phlegmonous and gangrenous appendicitis. However, both CRP (*p* = 0.045) and PCT (*p* = 0.005) medians were significantly higher in perforation/abscess compared to gangrenous appendicitis.

Complicated diverticulitis cases, compared to uncomplicated cases, demonstrated significantly and consistently higher LC (*p* = 0.007), NC (*p* = 0.01), and PCT (*p* = 0.048) median values, while CRP medians did not differ significantly (*p* = 0.199) between complicated and uncomplicated cases.

More severe cases of cholecystitis (gallstones without cholecystitis vs. mild vs. severe) exhibited significantly and consistently higher LC, NC, and CRP median values. Although the PCT median and distribution were visibly higher on a log-scale when comparing mild cholecystitis vs. gallstones without cholecystitis, the difference in medians was not significant (*p* = 0.065). In contrast, significantly higher PCT medians and distributions were found when comparing severe vs. mild cases (*p* = 0.0003). Notably, patients with pancreatitis (*n* = 21) were divided into mild (*n* = 18) and severe (*n* = 3) groups, and, as expected, inflammatory biomarker levels did not show significant differences in the severity grading of pancreatitis (*p* = 0.40).

**Fig. 3:**
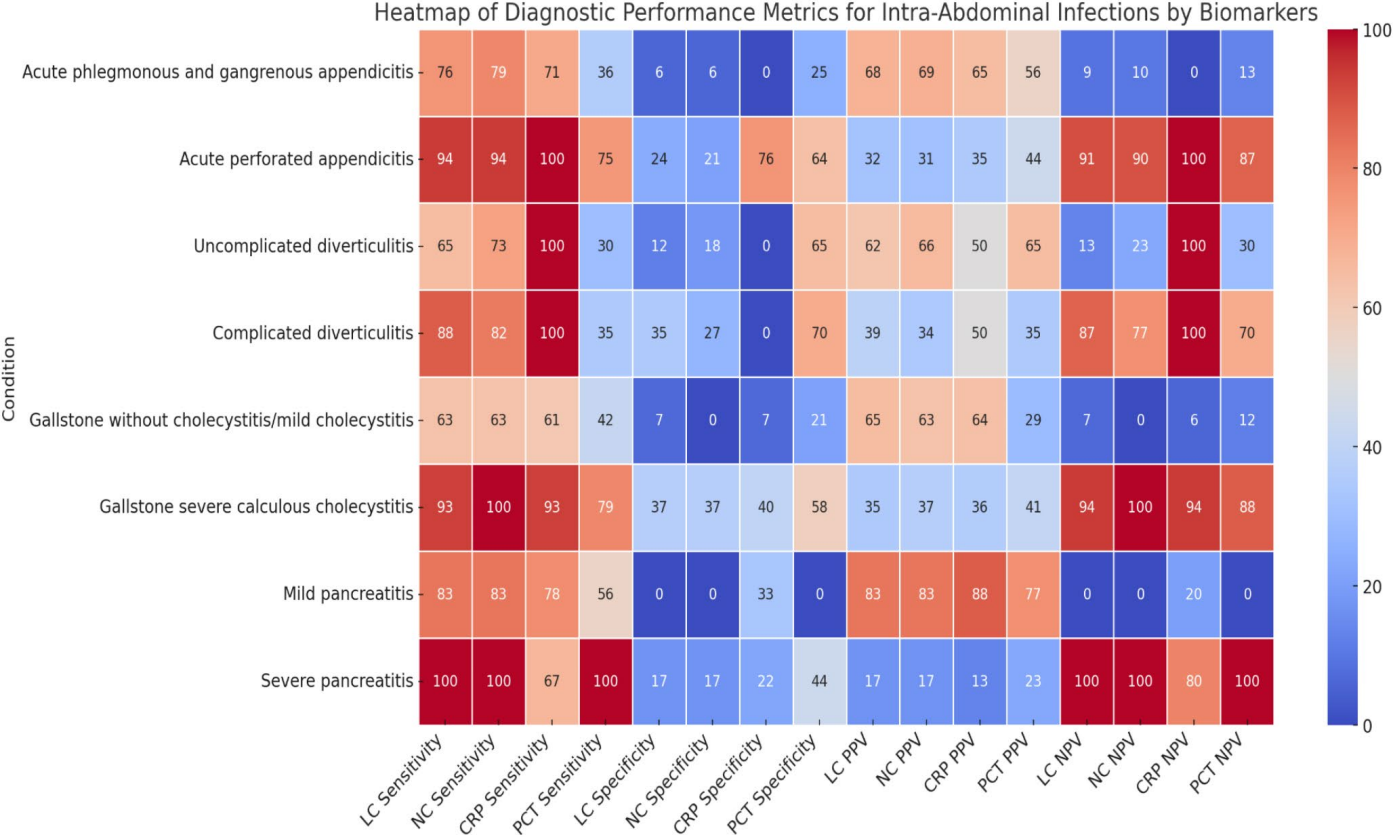
Comparison diagnostic performance metrics clinical severity of the intra-abdominal infections by biomarkers. The laboratory cut-off values of PCT ≥0.04 µg/L, LC (≥10.0 × 10^9^/L), NC (≥7.00 × 10^9^/L), and CRP (≥8.0 mg/L). Abbreviations: LC (leukocyte count); NC (neutrophil count); CRP (C-reactive protein); PCT (procalcitonin); PPV (positive predictive value); NPV (negative predictive value)

Figure 3 depicts the sensitivity, specificity, PPV, and NPV of inflammatory biomarkers. PCT performed poorly like other conventional inflammatory biomarkers for acute phlegmonous/gangrenous appendicitis, and the PCT accuracy was similar to that of other markers in acute perforated appendicitis. PCT has limited sensitivity in uncomplicated diverticulitis and should not be used alone for diagnosis. In complicated cases, its moderate negative predictive value can provide some reassurance if negative, but it should be used with other clinical assessments. Overall, PCT alone cannot reliably rule out diverticulitis. PCT is not highly effective in diagnosing mild calculous cholecystitis, so it should not be relied on as a standalone test. However, PCT is more effective in cases of severe calculous cholecystitis, making it a valuable component of the diagnostic process for more serious conditions. With moderate sensitivity, PCT can be used alongside other tests to enhance overall diagnostic accuracy, particularly in distinguishing between mild and severe cases. Mild pancreatitis shows that PCT is not very effective, with a low sensitivity. However, for severe pancreatitis, PCT is highly effective, demonstrating perfect sensitivity and NPV. In summary, PCT works better for diagnosing severe pancreatitis than for mild cases.

## Discussion

This study explored the utility of PCT in grading IAI clinical severity compared with a panel of conventional inflammatory biomarkers. From an IAI diagnosis-specific perspective, median PCT values were significantly higher in patients with cholecystitis and pancreatitis, but not in those with appendicitis and diverticulitis, compared with NSAP. The proportions of patients with PCT cut-off values ≥ 0.04 µg/L were significantly higher in all four IAI subgroups compared with NSAP. However, a surprisingly low proportion of patients with IAI exhibited PCT cut-off values ≥ 0.5 µg/L. A strong correlation exists between conventional inflammatory biomarkers and PCT in patients with IAI. In terms of severity grading, the median PCT values were significantly higher only in perforation/abscess vs. gangrenous appendicitis, complicated vs. uncomplicated diverticulitis, and severe vs. mild cholecystitis.

The results of this study provide valuable insights into the role of PCT and conventional inflammatory biomarkers in the diagnosis and severity assessment of IAIs. Our findings show that median PCT values were significantly higher in patients with calculous cholecystitis and pancreatitis compared to those with NSAP, but not in appendicitis and diverticulitis compared with NSAP. Additionally, a higher proportion of patients in all four IAI subgroups had PCT levels ≥ 0.04 µg/L compared with the NSAP group. Similarly, PCT levels ≥ 0.5 µg/L, considered the standard for bacterial infection, were statistically significantly higher, except in patients with diverticulitis. It is noteworthy that a low proportion of patients with IAI exhibited PCT values ≥ 0.5 µg/L, despite this cut-off being the accepted standard for bacterial infections. Importantly, PCT demonstrated a strong correlation with other established markers of inflammation, including LC, NC, and CRP.

Conventional biomarkers such as LC and NC significantly increase with the severity of acute appendicitis, diverticulitis, and cholecystitis, except pancreatitis, whereas CRP did not significantly increase in complicated diverticulitis or pancreatitis. Regarding severity grading, median PCT values were significantly higher only in perforation/abscess vs. gangrenous appendicitis, complicated vs. uncomplicated diverticulitis, and severe vs. mild cholecystitis, suggesting its potential utility as a severity indicator, except in pancreatitis severity grading. These findings support the potential of PCT as a valuable biomarker in assessing the severity of IAI, which could have important implications for decision-making.

### Appendicitis

In a systematic review by Joseph et al., PCT is highlighted as valuable for assessing appendicitis severity, especially in cases with perforation, gangrene, or necrosis [17]. Similarly, López et al. observed higher PCT levels in patients with complicated appendicitis [35]. Our study also found a significant difference in PCT levels, supporting its potential as a diagnostic tool for assessing the severity of acute appendicitis and guiding early antibiotic treatment. CRP is the most effective biomarker for diagnosing acute perforated appendicitis, with the highest sensitivity, specificity, and NPV. It is also valuable in acute phlegmonous appendicitis but lacks specificity.LC and NC provide better sensitivity than PCT in both types of appendicitis but also have low specificity. PCT is less effective in diagnosing phlegmonous cases but offers improved performance in perforated appendicitis.

### Diverticulitis

Victor et al. noted that combining PCT with abdominal CT scans could reduce antibiotic use by 80%, aiding clinical decision-making [21]. In our study, antibiotic treatment was not initiated in uncomplicated diverticulitis patients after diagnosis by CT scan, except in certain cases. Therefore, in our context, PCT evaluation should be aligned with CT scans for patients presenting to the emergency department to help determine whether to initiate antibiotics, rather than considering discontinuation once antibiotics have been started. Julide et al. reported higher PCT levels in more severe diverticulitis cases (Hinchey stages 3–4) [36]. Both Victor et al. and Julide et al. found that median PCT levels are useful in differentiating complicated from uncomplicated diverticulitis. Consistent with these findings, our study confirmed the statistical significance of PCT in differentiating between uncomplicated and complicated diverticulitis. However, PCT did not prove to be superior to already established biomarkers such as LC and NC. CRP is the most effective biomarker for diagnosing both uncomplicated and complicated diverticulitis, exhibiting high sensitivity and perfect NPV. LC and NC have better sensitivity than PCT in both types of diverticulitis, making them valuable in diagnosing these conditions, but they suffer from low specificity. PCT shows low sensitivity overall, particularly in uncomplicated cases, but has improved specificity in complicated diverticulitis, though it remains less effective compared to the other markers. In summary, for diverticulitis, particularly complicated cases, CRP is the preferred biomarker due to its diagnostic reliability, while LC and NC provide additional valuable information. PCT, while having some utility, is generally less effective in this setting.

### Cholecystitis

PCT is reported as a useful laboratory tool, similar to other biomarkers, in assessing the severity of acute cholecystitis [20]. In this study, a statistically significant increase in disease severity was detected with rising PCT levels in 95 patients with calculous cholecystitis. Wu et al. further supported PCT’s role by indicating that its elevation could serve as a preoperative risk assessment tool in acute cholecystitis patients [37]. In contrast, Yucel et al. found that PCT alone is not effective but could be considered an additional biomarker in the “Tokyo Guidelines [29]” for assessing the severity of acute cholecystitis [38]. In our study, PCT levels correlated with the severity of acute calculous cholecystitis. However, while differentiating between mild and severe forms of cholecystitis, our results suggest that PCT may not add significant value in determining the urgency and aggressiveness of antibiotic treatment or the timing of surgery. All biomarkers show limited effectiveness in diagnosing and ruling out the condition for mild calculous cholecystitis, with low sensitivity and specificity. For severe calculous cholecystitis, NC shows the highest sensitivity (100%), making it the most reliable marker for identifying severe cases. LC and CRP also perform well, while PCT provides a balance of sensitivity and specificity. PCT shows improved performance in severe cases compared to mild ones, but overall, LC and NC are more effective across different aspects of diagnosis for severe cholecystitis. In summary, LC and NC are preferred biomarkers for diagnosing severe calculous cholecystitis, while PCT has limited utility, particularly in mild cases. CRP also performs well in severe cases but lacks specificity in mild cholecystitis.

### Pancreatitis

PCT-guided care can reduce antibiotic use in acute pancreatitis without harm, according to Siriwardena et al. [39], and the reliability of PCT in assessing severity and predicting antibiotic requirements was validated by Alberti et al. [40] Additionally, Mann et al. suggested a role for PCT in antibiotic initiation, duration, risk of organ failure, and mortality prediction [41]. Conversely, Tarjan et al. found that the predictive value of PCT for early infections was limited but improved over time, especially in necrotizing pancreatitis [42]. In this meta-analysis, which included 13 studies, the severity of acute pancreatitis was evaluated through repeated PCT measurements after 48 h. However, in our study involving 21 pancreatitis patients, severity was assessed with a single measurement correlated with CT scan findings, indicating the need for repeated PCT measurements in future studies. In contrast, in our study, PCT was not significant for assessing severity or guiding early antibiotic use in pancreatitis. CRP is the most effective biomarker for diagnosing mild pancreatitis, showing good specificity and some sensitivity. However, none of the biomarkers perform well in ruling out the condition. In severe pancreatitis, PCT is the standout marker, demonstrating perfect sensitivity and high NPV, making it highly reliable for identifying severe cases. While CRP is useful, its lower sensitivity and specificity limit its utility compared to PCT. LC and NC are less effective overall for both mild and severe pancreatitis, showing low sensitivity in severe cases and limited specificity in mild cases. In summary, PCT is the preferred biomarker for diagnosing severe pancreatitis, while CRP provides some utility in mild cases. LC and NC do not significantly contribute to diagnosing pancreatitis effectively.

The main limitation of our data pertains to its retrospective observational nature, with potential confounders. Firstly, this was a single-center quality control study aimed at reporting our findings, making the results specific to our practice, and influenced by population characteristics. Furthermore, the smaller number of patients in the subgroups introduces the potential for type 1 error. Additionally, the absence of data on other clinical parameters (e.g., temperature, vital signs) and comorbidities limits a comprehensive understanding. Another limitation is the lack of blood culture data, which could have provided additional confirmation of bacterial infection alongside PCT levels.

Despite these inherent limitations, our retrospective single-center study provides valuable insights based on a robust real-world experience involving a regional cohort of unselected acute abdomen patients with precise bio-clinical data. We employed well-established methodologies, enhancing the reliability of our findings. To strengthen the validity of our results, we exclusively examined primary final diagnoses, ensuring that hospital admissions were directly attributable to our specified endpoints rather than being influenced by prior registry records or secondary complications. Notably, the inclusive nature of our data, encompassing all residents of the region regardless of labor market participation, minimizes selection bias related to specific age groups or insurance systems, thereby bolstering the generalizability of our results.

In conclusion, the study demonstrated that PCT is strongly correlated with conventional inflammatory biomarkers in patients with IAI. Combining CRP and PCT can improve diagnostic accuracy by balancing sensitivity and specificity. While CRP is highly sensitive in detecting serious conditions such as acute perforated appendicitis, complicated diverticulitis, and severe pancreatitis. PCT has higher specificity, aiding in ruling out other conditions in different situations. However, PCT appears to provide limited additional clinical value in guiding therapy decisions regarding the initial diagnosis and/or severity grading of IAI for patients admitted with acute abdomen in emergency settings. Further research with larger and more diverse patient populations is warranted to validate our observations and better understand the real value and clinical utility of PCT in managing acute abdomen.

## Data Availability

No datasets were generated or analysed during the current study.
